# Acute appendicitis: proposal of a new comprehensive grading system based on clinical, imaging and laparoscopic findings

**DOI:** 10.1186/s13017-015-0053-2

**Published:** 2015-12-03

**Authors:** Carlos Augusto Gomes, Massimo Sartelli, Salomone Di Saverio, Luca Ansaloni, Fausto Catena, Federico Coccolini, Kenji Inaba, Demetrios Demetriades, Felipe Couto Gomes, Camila Couto Gomes

**Affiliations:** Surgery Department, Therezinha de Jesus University Hospital, Medical and Health Science School, Surgery Unit, Federal University of Juiz de Fora (UFJF), Rua Senador Salgado Filho 510 / 1002, Bairro Bom Pastor, Juiz de Fora, Minas Gerais 36021-660 Brasil; Department of Surgery, Macerata Hospital, Macerata, Italy; Trauma Surgery Unit, Maggiore Hospital, Bologna, Italy; General Surgery I, Papa Giovanni XXIII Hospital, Bergamo, Italy; Emergency Surgery Department, Maggiore Parma Hospital, Parma, Italy; University of California, San Francisco, USA; Department of Surgery (K.I.), Keck School of Medicine of University of Southern California, Los Angeles, CA USA; Internal Medicine Departament, Therezinha de Jesus University Hospital, Medical and Health Science School, Juiz de Fora, Brazil; Internal Medicine Departament, Monte Sinai Hospital, Juiz de Fora, Minas Gerais Brazil

**Keywords:** Appendicitis, Appendectomy, Laparoscopy, Treatment, Classification

## Abstract

Advances in the technology and improved access to imaging modalities such as Computed Tomography and laparoscopy have changed the contemporary diagnostic and management of acute appendicitis. Complicated appendicitis (phlegmon, abscess and/ or diffuse peritonitis), is now reliably distinguished from uncomplicated cases. Therefore, a new comprehensive grading system for acute appendicitis is necessary. The goal is review and update the laparoscopic grading system of acute appendicitis and to provide a new standardized classification system to allow more uniform patient stratification. During the last World Society of Emergency Surgery Congress in Israel (July, 2015), a panel involving Acute Appendicitis Experts and the author’s discussed many current aspects about the acute appendicitis between then, it will be submitted a new comprehensive disease grading system. It was idealized based on three aspect of the disease (clinical and imaging presentation and laparoscopic findings). The new grading system may provide a standardized system to allow more uniform patient stratification for appendicitis research. In addition, may aid in determining optimal management according to grade. Lastly, what we want is to draw a multicenter observational study within the World Society of Emergency Surgery (WSES) based on this design.

## Background

Appendicitis is the most common cause of an acute surgical abdomen, with an estimated lifetime prevalence of 7–8 %. Despite advances in diagnosis and treatment, it is still associated with significant morbidity (10 %) and mortality (1–5 %) [1]. The clinical history and physical examination represent the most important tools for early diagnosis of the disease. The overall accuracy for diagnosing acute appendicitis is approximately 90 %, with a false-negative appendectomy rate of 10 %. This is more frequent in atypical cases, especially in women of childbearing age, because the symptoms often overlap with others conditions [2, 3]. Recently 182 patients with suspicion of acute appendicitis were stratified to low, intermediate, and high probability of appendicitis by two different clinical scores (AIR / Alvarado) and by an experienced surgeon. The AIR score was especially good at identifying patients with high probability of appendicitis with a specificity of 0.97 for all appendicitis and 0.92 for advanced appendicitis, compared with 0.91 and 0.77, respectively, for the surgeon and Alvarado score. Therefore, in this series, the AIR score had both higher sensitivity and specificity than the Alvarado score and the experienced surgeon in the clinical diagnosis of the disease [4].

## Imaging

The clinical scores represent an excellent and useful tool for pre-operative diagnosis of acute appendicitis, but regardless its accuracy it cannot be applied as a grading system for acute appendicitis, especially attempting to distinguish different complicated grades of the disease [5]. As we know, novel scoring systems are being described and introduced into clinical practice, based on clinical and imaging (CT and/or US). In addition, less invasive management options including percutaneous drainage, non-operative treatment and minimally invasive surgery are available [6].

Three imaging modalities are available in difficult cases of acute appendicitis: Ultrasound (US), Computed Tomography (CT), and Magnetic Resonance Imaging (MRI). Trans-abdominal ultrasound should be the first-line imaging test. Although there is a higher radiation burden, abdominal CT is superior to US and may be required in patients with an equivocal US or if perforation is suspected. Low-dose unenhanced CT is equivalent to standard-dose CT with intravenous contrast agents in the detection of the five signs of acute appendicitis (thickened appendiceal wall greater than 2 mm, cross-sectional diameter greater than 6 mm, increased pericolic fat density, abscess, and appendicolith) [7]. However, as pointed out by Saar, despite all available technologies, it remains very difficult to achieve a false negative appendectomy rates less than 10 % [8].

## Operative versus non-operative treatment

Both open appendectomy and laparoscopic appendectomy are acceptable techniques and can be used interchangeably. The laparoscopic treatment of uncomplicated grades of acute appendicitis is well established and represent the approach of first choice some time ago. However, well-conducted trials to help guide the treatment for all complicated grades of acute appendicitis are limited, especially by the presence of bias and methodological flaws. However, the safety and efficacy of laparoscopy in the treatment of these cases is well established too [9–13].

A recent meta-analysis by Varadhan et al. 2015 [14] assessed four randomized controlled trials about safety and efficacy of antibiotics compared with appendectomy for treatment of uncomplicated acute appendicitis [15–18]. The primary outcome measure was the incidence of complications and secondary outcome was the efficacy of treatment. 900 patients (470 antibiotic treatment, 430 appendectomy) met the inclusion criteria. Antibiotic treatment was associated with a 63 % (277/438) success rate at 1 year. Meta-analysis of complications showed a relative risk reduction of 31 % for antibiotic treatment compared with appendectomy. The authors concluded that antibiotics are both effective and safe as primary treatment for patients with uncomplicated acute appendicitis. Initial antibiotic treatment deserves consideration as a primary treatment option for early-uncomplicated appendicitis.

Similarly, the study NOTA (Non Operative Treatment for Acute Appendicitis), assessed the safety and efficacy of antibiotic treatment for suspected acute uncomplicated appendicitis and monitored the long-term follow-up of non-operated patients. One hundred fifty-nine patients with suspected appendicitis were enrolled and underwent non-operative management with amoxicillin / clavulanate. The follow-up period was 2 years. Short-term (7 days) non-operative failure rate was 11.9 %. All patients with initial failures were operated within 7 days. At 15 days, no recurrences were recorded. After 2 years, the overall recurrence rate was 13.8 %. The authors concluded that antibiotics for suspected acute appendicitis are safe and effective and may avoid unnecessary appendectomy, reducing operation rate, surgical risks, and overall costs [19].

Although interesting and reducing the false negative appendectomy rate, both studies also contain methodological flaws, like the patients recruitment, surgery approach (laparotomy/laparoscopy), different antibiotics prescription and images diagnostic method criteria (CT scan / Ultrasound). In addition, the success rate of 63 % is very low and the relative risk of complication reduction very high. Therefore, the laparoscopic treatment of non-complicated acute appendicitis may show much less complication rates and represent the treatment of choice with acceptable false negative appendectomy rate about 10 % [11, 20].

## Histologic diagnosis

As a rule, the acute appendicitis diagnosis was established according to the transmural appendix inflammation (neutrophilic infiltration of the mucosa, submucosa, and *muscularis propria*). The histologic assessment also defined the difference between endoappendicitis (neutrophils within mucosa and mucosal ulceration) and periappendicitis (inflammation restricted to serosa and sub-serosa) [21].

### Why to propose a new acute appendicitis grading system?

The laparoscopic grading system of acute appendicitis proposed by Gomes et al. [20] is limited by its exclusive focus on just the intraoperative aspects (Table 1). Complicated grades (phlegmon, abscess and/or diffuse peritonitis), are now reliably distinguished from uncomplicated cases by clinical and imaging findings. Because the treatment options for these complicated cases of acute appendicitis includes non-operative modalities, a new comprehensive grading system for acute appendicitis is necessary (Table 2).

**Table 2:** Proposal of a new grading system of acute appendicitis based on clinical, imaging and laparoscopic findings (2015)

Non-Complicated Acute Appendicitis	
Grade 0 - Normal Looking Appendix (Endoappendicitis/Periappendicitis).	
Grade 1 - Inflamed Appendix (Hyperemia, edema ± fibrin without or little pericolic fluid).	
Complicated Acute Appendicitis	
Grade 2 – Necrosis	A - Segmental Necrosis. (without or little pericolic fluid).
	B - Base Necrosis. (without or little pericolic fluid).
Grade 3 - Inflammatory Tumor-	A Flegmom.
	B - Abscess less 5 cm without peritoneal free air.
	C - Abscess above 5 cm without peritoneal free air.
Grade 4 - Perforated - Diffuse Peritonitis with or without peritoneal free air.	

Note: Proposal for a new acute appendicitis grading system based on clinical, imaging and laparoscopic findings. (±) = Presence or absence of fibrinous exudate Gomes et al. (2015).

It was idealized a grading system for acute appendicitis that incorporates clinical presentation, imaging and laparoscopic findings. The goal of this new grading system is to provide a standardized classification to allow more uniform patient stratification for appendicitis research and to aid in determining optimal management according to grade (Table 2).

**Table 1:** Laparoscopic grading system of acute appendicitis

Grade	Laparoscopic findings
Grade 0	Normal looking appendix
Grade 1	Hyperemia and edema
Grade 2	Fibrinous exudate
Grade 3A	Segmental necrosis
Grade 3B	Base necrosis
Grade 4A	Abscess
Grade 4B	Regional Peritonitis
Grade 5	Difuse Peritonitis

Note: From Gomes et al. 2012 [13]

## New acute appendicitis grading system

### Grades

#### Grade- 0 (normal looking)

**Fig. 1:**
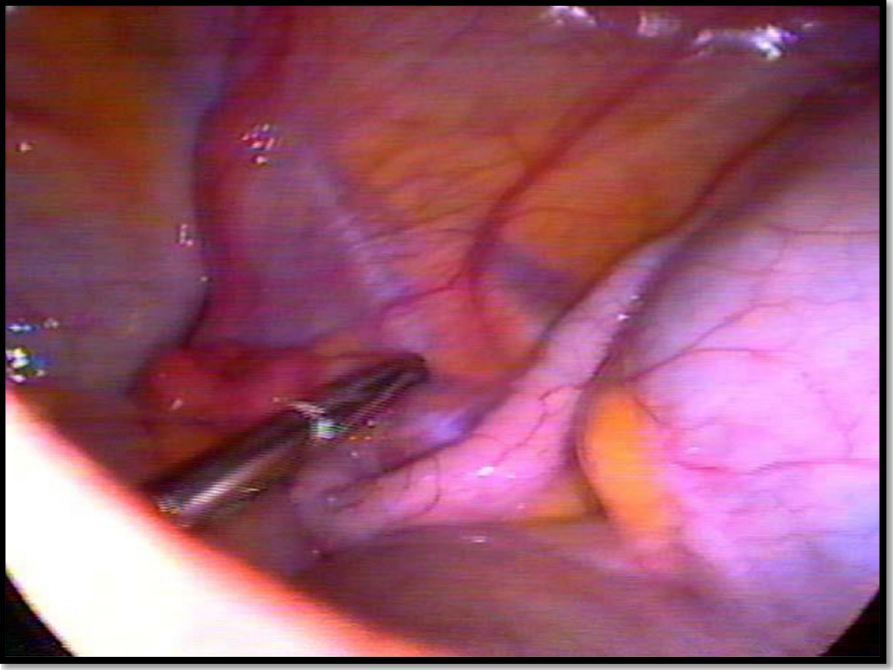
“Normal looking appendix” without any other intra-abdominal disease and suggestive clinical set. The appendix was removed and the histopathological study shows intraluminal inflammation (Grade 0)

The grade 0 refers to the non-rare situation surgeon may faces, when the patient has a clinical diagnosis of acute appendicitis and laparoscopy shows a macroscopically “normal looking appendix”. In such case, if the appendix looks normal on laparoscopy but another disease is found to be the cause of the patient’s symptom, then the appendix should be left in situ [22]. The 10-year follow-up by van Dalen et al. [23], demonstrated the safety of this approach in women. The situation is more complicated when the appendix shows no signs of inflammation and no other disease can be found (Fig. 1). Weighting the disadvantages of a negative appendectomy against the risk of overestimating a case of appendicitis is difficult. If symptoms and signs are typical for appendicitis, most surgeons still consider advised to perform an appendectomy, because in early appendicitis, the inflammation may be limited to intramural layers [11].

In surgical cases of pelvic endometriosis, surgeons need to preoperatively inform that appendix is found frequently involved, regardless the presence of concurrent symptoms or gross finding of the appendix. Furthermore, surgeons should take into account the possibility of performing an incidental appendectomy [24].

#### Grade-1 (inflamed)

**Fig. 2:**
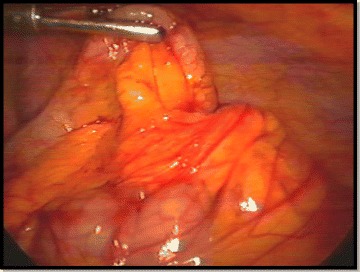
The image shows hyperemia and edema of appendix, without fibrinous exudate (Grade-1)

Gomes et al. in 2012, published a series of 186 patients who underwent a laparoscopic appendectomy, according to the Laparoscopic Grading System for Acute Appendicitis (Table 1). This grading system has been developed to stratify the disease according to the inflammatory findings occurring within the appendix and the abdominal cavity. The impact of the grade on surgical site infection was also examined [20]. This score was externally validated in a cohort of 112 consecutive cases of complicated acute appendicitis patients by Di Saverio et al, where all patients had Gomes scores II–V and the scores were correlated with the outcomes [25]. Based on this series the safety and efficacy of laparoscopy compared to open appendectomy was also examined. The laparoscopic grading system was useful in stratify the disease; contributing and highlights some aspects, whose laparotomy could not be able to show at the same amplitude (Fig. 2) [20].

In addition, Gomes et al. documented an unusual situation. About 10 % of the patients where appendix presented with hyperemia, edema and fibrin exudates had a significant plasma exudation into the abdominal cavity. The study of the exudates diagnosed the presence of gram-negative bacteria in 10 % of the analyzed samples. These data could explain, at least partially, that acute appendicitis may get complicated with development of postoperative peritonitis and intra-abdominal abscesses after simple appendectomies, especially when antimicrobial prophylaxis was not administrated. Excessive plasma exudation in the absence of necrosis and/or perforation of resected appendices could be explained by bacterial translocation and plasma transudation [20].

#### Grade- 2A and 2B (necrosis)

**Fig. 3:**
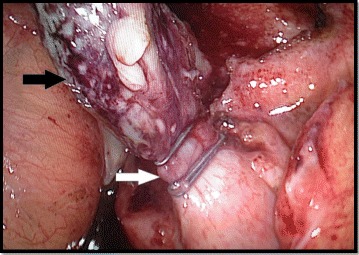
Acute appendicitis with segmental necrosis (*black arrow*). Observe that the appendiceal base was not compromised by inflammatory and necrotic process, allowing appendicular stump closure by metallic clip (*white arrow*) In a healthy tissue (Grade 2A)

Complicated appendicitis refers to gangrenous and/or perforated appendix, which may lead to abscess formation and degrees of peritonitis [26]. Therefore, these grades by definition are complicated cases of acute appendicitis. Nevertheless, the specific grade study, showed that in the grade 2A, the necrosis was an isolated phenomenon, restricted to the appendix, without or with minimal local exudation (Fig. 3). The majority of patients had an uneventful recovery and were discharged in the next postoperative day. More importantly, they had a clinical course similar to those with non-complicated appendicitis (grade 0, 1). They received short course antimicrobial therapy (3 to 5 days) and post-operative complication was a rare event. By the way, recent observational cohort study from van Rossem et al. showed that after appendectomy for complicated appendicitis, 3 days of antibiotic treatment is equally effective as 5 days in reducing postoperative infections [27].

About 3.2 % there was presence of necrosis involving the appendicular base, at the level of its insertion on cecal wall (grade 2B). This condition makes the operation even more difficult and requires experience from the surgical team with intra-corporeal suturing, mainly when endostapler is not routinely used, justifying a new specific grade, which is rarely studied during laparoscopic appendectomy. Nowadays, this grade represents the most important situation, where the endostapler is used to closure the appendiceal stump in the Service. In the other grades the appendicular stump could be closure of different ways (endostapler, endoloop, metallic and polymeric clip and others one). We prefer its management by T-400 metallic endoclip, which is less expensive and have demonstrated safety and effectiveness in a prospective observational study [20]. In addition, it is oriented operating the patients under Day Hospital way. The study of Alvarez and Voitk [28], should be highlighted because, according the authors, in the ambulatory management of acute appendicitis (Day Hospital), the patients discharge is occurring less than 24 h after appendectomy and this recommendation was adopted for grades 0,1, 2 [28].

#### Grade- 3A - 3B - 3C (perforated - inflammatory tumor)

**Fig. 4:**
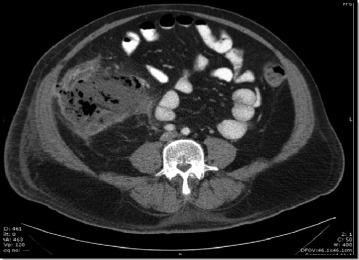
The CT scan of abdomen showing an inflammatory tumor in the lower right quadrant. The patient was managed with antibiotics only; i. e non-operative treatment. (Grade 3A)

**Fig. 5:**
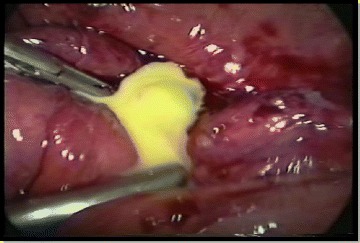
Acute appendicitis complicated with inflammatory tumor and an abscess less than 5 cm, managed by laparoscopic approach (Grade 3B)

As it is already well known, sometimes the inflammation of the appendix may be enclosed by the patient’s own defense mechanisms, by the formation of an inflammatory phlegmon or a circumscribed abscess of different diameter, often presenting some days after the onset of symptoms [29]. In fact, an inflammatory tumor in the right lower quadrant represents a spectrum, at least of three physiopathology stages of the acute appendicitis, very similar to what happens in the acute diverticulitis of sigmoid colon: phlegmon, inflammatory tumor with <5-cm abscesses and inflammatory tumor >5-cm abscess (Fig. 4). Thus, once again, such patients should not be considered as a whole, without distinction, since they have different aspects with regard to, physiopathology, treatment, complications, disease recurrence and prognosis. Moreover, according to Stefanidis et al 2008, acute abdominal pain lasting less than 7 days [30]. Therefore, assuming that we are evaluating patients with acute and subacute disease, since mostly patients classified within these grades, had the onset of symptoms occurring into seven or more days. These patients receive long course (5–10 days) antimicrobial therapy according their clinical post-operative recovery (Fig. 5).

#### Grade- 4 (perforate - diffuse peritonitis)

**Fig. 6:**
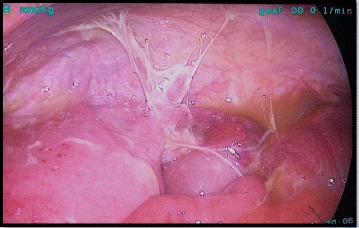
Patient with perforated appendicitis and diffuse peritonitis operated on laparoscopy. He had an uneventful recovery in the postoperative day six. (Grade 4)

Controversy exists regarding the laparoscopic approach in the treatment of acute appendicitis complicated with diffuse peritonitis. The chance of potential surgical complications is high and consequently the outcomes are poorly documented. Our literature review found only two articles investigating the issue [31, 32]. Although the results seems to favor the use of laparoscopy, only a large multi-institutional study with appropriate design will be able to answer this question (Fig. 6).

## Summary

In summary, the new appendicitis grading system is based on three aspects of the disease. The clinical, imaging and laparoscopic findings and could be tested in multicenter observational study within the World Society of Emergency Surgery, in order to assess its actual practicality. It will enable the creation of homogeneous groups of patients with disease in the same well-defined stage. Ultimately, the goal of this grading system is to aid in determining optimal management according to grade, and to provide a standardized classification system to allow more uniform patient stratification for appendicitis research.
